# The ‘R’ principles in laboratory animal experiments

**DOI:** 10.1186/s42826-020-00078-6

**Published:** 2020-12-09

**Authors:** Kook Hyun Lee, Dong Won Lee, Byeong Chul Kang

**Affiliations:** 1 Department of Anesthesiology, Seoul National University Hospital, 101 DaeHak-ro, Jongno–Gu, Seoul, 03080 South Korea; 2 Department of Clinical Research Institute, Seoul National University Hospital, Seoul, South Korea

**Keywords:** Replacement, Reduction, Refinement, Responsibility, Refusal, Welfare

## Abstract

Since the Three Rs of replacement, reduction and refinement was proposed by Russel and Birch in 1959, researchers have a moral duty to minimize harm to animals. Even though animal experiments are performed by the Three Rs concept, animal researches which do not comply with international rules and standards are not accepted as well. As animal welfare has been important global issues, the methods to assess animal welfare compromise and distress have been proposed. Humanity is accepted as the goal of the Three Rs, however, another fourth R, ‘Refusal’ of fruitless protocol or ‘Responsibility’ for the experimental animal and social, scientific status of the animal experiments has been proposed. After establishing goals of animal research in a respective society, reliable knowledge can be obtained while improving laboratory animal welfare.

## Introduction

Though humans are phylogenetically at the peak of the evolutionary stage, it seems unlikely that human evolution is the best in all respects because animal evolution has moved toward optimizing for the environment. In recent years, the respiratory tract viruses which transmit Influenza and Coronavirus disease, choose animals and humans as hosts without any difference, and it is a reality that human bodies also contribute to the evolution of pathogens. However, the concern for the welfare of animals can be said to be the only characteristic that mankind is different from other animal species. Since the Three Rs of replacement, reduction and refinement was proposed by Russel and Birch in 1959 [1], ‘refusal’ or ‘responsibility’ as the fourth R, has been proposed to improve the scientific benefit while minimizing harm to laboratory animals.

## Main text

Ethics is defined as ‘a branch of philosophy that involves systematizing, defending, and recommending concepts of right and wrong behavior’ [2]. However, ethics is acknowledged as a principle that becomes the norm of actual morality. Similar to the Ethics Committee deliberating experimental medical treatments such as organ transplant in human hospitals, the Institutional Animal Care and Use Committee (IACUC) is established to review studies on laboratory animals. Principles of animal testing basically considers both the welfare of humanity and the dignity of animal life. In addition to ethics, the experimental protocol review is conducted from the viewpoint of the scientific benefit versus cost of laboratory resources.

After the Three Rs of Russel and Birch in 1959 were introduced, replacement has been considered prior to reduction with refinement being considered last [3]. Even though animal experiments are performed by replacing animals with computer models, tissue or cell cultures while reducing the number of animals for the valuable knowledge by less harmful practices, it is true that the public watch the animal experiments seriously and the researches which do not comply with international rules and guidelines are not accepted as well. However, many of the proposals without external fund submitted to the IACUC should be evaluated cautiously from the aspect of animal well-being and scientific gain. Curzer et al. [3] proposes “Refusal” as another R, for the IACUC to reject the protocol when knowledge gain is unjustified at the cost of harm to animals which cannot be clarified well. It can be accepted that refusal or rejection might result from the meticulous application of replacement or refinement during the early protocol review of the IACUC. Of note is that “Refusal “can be an action plan to enhance the Three Rs principles, resulting in replacing some of in-vivo experiments with in-vitro tests while referring to other alternative methods. However, the “Refusal” implies that animals may not be used without a reasonable benefit.

The knowledge gained through the experiment is a benefit to society while researchers have a moral duty to minimize the harms to animals. It has been admitted that using animals in research aims at improving human health and well-being. The American Medical Association has proposed humane care and well-being of laboratory animals, since animal testing is required to treat immunodeficiency virus diseases, cancer, heart disease, dementia, stroke, congenital malformations and developmental disorders [4].

After the five domains model, which includes four domains of nutrition, environment, health and behavior, and a fifth of mental state [5] was developed to assess welfare compromise in sentient animals, World organization for animal health [6] provided the internationally recognized ‘five freedoms’ as guiding principles for animal welfare. The welfare of laboratory animals might be different from that of wild or companion animals. However, humane animal care and laboratory environment should be provided well because laboratory animal welfare is also related to the reliable research data.

As animal welfare had been important global issues, pain and distress are recognized as important factors affecting animal welfare. Measuring distress can be very difficult and failure to avoid distress may adversely affect scientific outcome resulting in the use of more animals than necessary. Biological responses to non-pain distress can be frequently observed in animals awaiting long time before the beginning of an experiment. The animal care staff, the researcher, veterinarians, and the IACUC should continue to monitor animals for pain, distress and illness during the research period.

The PREPARE (Planning Research and Experimental Procedures on Animals: Recommendations for Excellence) checklist suggests that the quality of scientific result is dependent upon planning and conducting [7]. In addition, transparent reporting of research findings is essential for the reproducibility of animal study, resulting in the realization of the scientific benefits to society as the ARRIVE (Animal Research: Reporting of In Vivo Experiments) guidelines suggest [8]. While adhering to ARRIVE recommended items, ethical review and realization of the Three Rs principles can be evaluated [9].

Previously, another “R” of responsibility was proposed for the experimental animal and social, scientific status of the animal experiments. It was emphasized that humane management of experimental animals and accountability for society are more important than the stance that animal experiments themselves are unethical [10]. In addition, “responsibility” can be interpreted as an essential concept for the researches to survive in a society irrespective of the experimental subjects. The public and research societies admit that researchers, IACUC and animal staff are all allowed to care and use live animals for the benefits of both humans and animals. Therefore, it can be recommended that the research mind of responsibility should be kept under the application of the principles of Three Rs for the fruitful in-vivo experiments [11].

Since the Three Rs become the norm of experimental practice, compliance with the law is essential for animal experiments. Though the ethical understanding of the principles might be somewhat different from the legal interpretation, animal experimentation should be morally permissible to help researchers modify an initial research plan. Many researchers become conscious of laboratory animal welfare under the legal environment and IACUC oversight. Laboratory animal technicians must play an important role in the everyday practice of animal care. Animals should be observed closely and provided with better housing condition, so that scientifically beneficial experiments may be performed with sound animal health.

## Conclusions

Each society has different philosophy on animal experiment, however, international principles can be regarded as the global value. After establishing goals of animal research in each respective society, reliable knowledge can be obtained while improving laboratory animal welfare. Humanity is accepted as the goal of the Three Rs concept. While maintaining the principles, refusal or responsibility spirit with humane practice can promote the health and well-being of humans and animals with appreciation for experimental animals sacrificed for bioscience.

## Data Availability

Not applicable. This manuscript is a brief report which reviews previous scientific reports while expecting future perspectives for laboratory animal welfare in Korea.

## Abbreviations

Three Rs: Replacement, reduction and refinement
IACUC: Institutional Animal Care and Use Committee
